# Molecular and phenotypic characterization of SG9R-like *Salmonella* Gallinarum field isolates from South Korea, 2019–2025

**DOI:** 10.3389/fvets.2026.1850190

**Published:** 2026-06-09

**Authors:** Van Dam Lai, Hoang Minh Nguyen, Hyunwoo Baek, Jongsuk Mo

**Affiliations:** 1 Department of Research, Avinext Ltd., Cheongju, Republic of Korea; 2 College of Pharmacy, Yeungnam University, Gyeongsan-si, Republic of Korea

**Keywords:** *aceE*, fowl typhoid, mutations, *rfaJ*, SG9R

## Abstract

*Salmonella enterica* serovar Gallinarum (SG) is a well-known bacterial pathogen in poultry as the causative agent of fowl typhoid (FT) fever. SG9R live vaccines has been widely used to control outbreaks of SG in South Korea for over 20 years. Recently, molecular analysis of SG9R field strains isolated between 2019 ~ 2025 from South Korea have revealed several mutations in key genes (*aceE, rfaJ*) associated with SG-associated markers. Despite being designated as SG9R in differential multiplex PCR assays, these unique isolates harbored mutations in the molecular markers of *aceE* and *rfaJ* genes which resemble those of SG strains. We also discovered these isolates resemble the phenotypic characteristics of the SG strains. Our study emphasizes the importance of monitoring and developing a new diagnostic approach to differentiate SG, SG9R and these unique strains (SG9R-like) thereby improving disease control strategies and minimizing economic losses in the poultry industry.

## 1 Introduction

*Salmonella enterica* serovar Gallinarum (SG) is considered a major poultry disease globally, capable of inflicting significant economic losses. As a well-known causative agent of fowl typhoid (FT), the disease is known to cause a wide range of clinical symptoms, such as anorexia, dehydration, decrease in egg laying rate and high mortality (1). While SG is not usually considered a major disease in European and North American countries due to implementation of strict biosecurity measures, it is still common in many developing countries in South America, Africa and Asia (2). In South Korea, since the first documented outbreak of FT in 1992, SG persists to this day, albeit outbreaks tend to be sporadic rather than developing into full-scale endemics. Field associated genetic changes in SG is relatively well studied, with focus on antibiotic resistance and epidemiology (2, 3). To effectively control SG outbreaks in the Korean poultry industry, SG9R vaccines were widely implemented and still the only commercial vaccine strain available in the country (4). The SG9R vaccine strain, while genetically similar to the wild-type SG strain, carries a mutation in the lipopolysaccharide gene (*rfaJ*) which likely functions as an attenuation marker (5). It is well known the TAA (Stop) mutation in the *rfaJ* may result in incomplete translation of the LPS core, thus resulting in contribution of attenuation (6). Several studies have reported that SG field isolates may be genetically related to SG9R vaccine strains (6–8). Previous investigations of *Salmonella* strains were usually focused on the pyruvate dehydrogenase (*aceE*) and the lipopolysaccharide 1,2-glucosyltransferase (*rfaJ*) genes, associated with attenuation and virulence-related phenotypes (4, 9). Recent FT field cases in Korea have shown chickens exhibiting clinical signs and necropsy findings similar to that of SG but diagnosed as SG9R by differential multiplex PCR. In this study, such isolates are referred to as SG9R-like isolates. SG9R-like isolates were detected by utilizing a differential multiplex PCR system, developed by the Korean Animal and Plant Quarantine Agency (QIA) which is widely used for diagnosis and differentiating SG and SG9R strains (10). As the SG9R vaccine strains has been use in Korea for over 20 years, we have hypothesized that changes in attenuation-associated markers (substitution sites in *aceE, rfaJ, spvB*) may have occurred in some SG9R-like field isolates, potentially contributing to the emergence of isolates with SG-associated molecular and phenotypic characteristics. In this study, we focused on the *aceE, rfaJ,* and *spvB* genes, of the recent Korean SG and SG9R-like isolates and analyzed the genetic markers which may have played a role in SG-associated marker restoration in the SG9R-like field isolates.

## 2 Materials and methods

### 2.1 Necropsy and bacteria isolation

A total of 138 field cases suspected of FT were submitted to Avinext LTD between 2019 and 2025 for necropsy. Cases were initially selected based on farm history, flock signalment, reported clinical signs or mortality, gross lesions observed by farm veterinarians, and suspicion of FT at the time of submission. Only cases confirmed as either SG or SG9R by differential multiplex PCR were included in the final dataset. Necropsy was conducted on dead birds using standard necropsy procedures. Carcasses were examined for gross lesions and multiple organ samples including trachea, liver, kidney and cecal tonsils were collected for PCR and bacteriology. Cases suspected of viral co-infections were submitted for PCR, using primers and cycling conditions as previously described by the Korean Animal and Plant Quarantine Agency (QIA) (11). Bacteria culture was conducted as previously described as per instructions provided by the QIA manual. To explain briefly, bacterial samples were first collected from tissues using a platinum wire hook and were streaked on MacConkey agar plates (Kasan Bio Co, South Korea) and grown for 24 h at 37 °C. Once presence of colorless colonies was confirmed, the colonies were re-propagated in TSB media (Kasan Bio Co, South Korea) overnight and sub-cultured in MacConkey agar. After sub-culture, colonies were selected and multiplex PCR were conducted according to QIA guidelines to differentiate between SG and SG9R strains (10, 11).

### 2.2 Sequencing and genetic analysis

Sequencing of the strains were conducted using specific primers targeting the virulence markers of the *rfaJ* and *spvB* genes with cycling conditions as previously described (4). Sequencing primers for *aceE* were designed in-house. To explain in detail, primer set (rfaJ-F 5'-cagttacgttatgcggcaaagc-3', rfaJ-R 5'-atacaactgaagcgattgatacc-3') were used for sequencing of rfaJ to produce a band product of 264 bp including the virulence-associated marker. Similarly, primer sets (spvBproF-5'-ctatgaaggcgacggttata-3', spvBproR-5'- attcctctacaatcgcccat-3') and primer set (AceE-F 5'- gtatctacagcccgaacggc-3', AceE-R 5' gacgcaggatagaaccggag-3') was used to sequence the proline repeats in the spvB (124 bp) and the partial ACE2 segment (608 bp), respectively. Cycling conditions were all identical for all reactions (94 °C, 5 min; 35 cycles at 94 °C, 30 s;55 °C, 30 s; and 72 °C, 2.5 min; and a final extension step at 72 °C, 5 min) Amplified PCR products were excised and purified via the Expin™ cleanup kit (GeneAll, Seoul, South Korea) according to manufacturer’s instructions. The purified products were submitted to Cosmogenetech (Seoul, South Korea) for sanger sequencing. Sequences were aligned and SNPs were analyzed via the Qiagen CLC genomic workbench software (Qiagen, Hilden, Germany). Sequence chromatograms were visually inspected base by base using the Finch TV software (Seattle, WA, United States) to evaluate base-call quality. If ambiguous base calls or low-quality peaks were present in the target marker positions, sequencing was repeated before assigning the final SNP profile.

### 2.3 Strain phenotyping and O:9 antigen assay

For phenotyping, propagated colonies were selected and mixed with 35 μL of 1X PBS and were applied on a clean microscope slide. Afterwards, 35 μL of 0.2% acriflavine (Sigma-Aldrich Korea, Seoul, South Korea) was added and gently mixed. After 30s, the mixture was checked for signs of agglutination. Agglutination was defined as the visible formation of grain-like structures in the mixture when observed with the naked eye. To improve consistency of interpretation, each result was independently confirmed by at least two personnel before assigning classification. Strains that agglutinated were classified as rough (R) strains and non-agglutinating strains were classified as smooth (S) strains. The O:9 antigen assay was conducted using the Difco DB O antigen kit (Franklin Lakes, NJ, United States) according to manufacturer’s instructions.

## 3 Results

### 3.1 SG9R field cases associated with pathogenicity in chickens have gradually increased between 2019 ~ 2025

**Figure 1:**
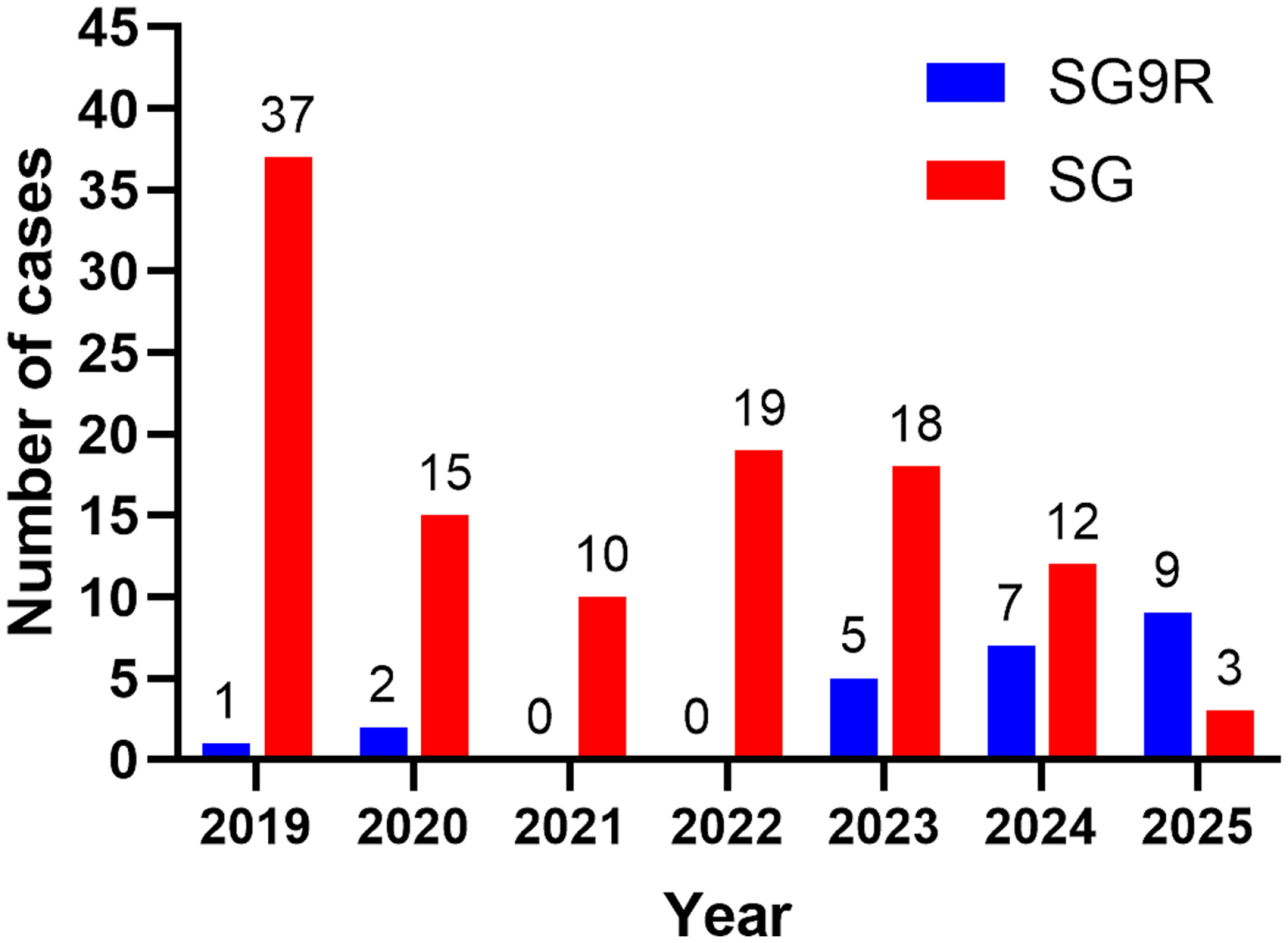
SG and SG9R confirmed cases by multiplex PCR assay. Number of cases are listed by year (2019–2025) and categorized into either SG or SG9R based on the screening PCR results. Out of total 138 cases, 24 cases were categorized as SG9R.

Out of 138 FT suspected cases, 24 cases were diagnosed as SG9R, representing 17.4% of all FT related cases (Figure 1). Details such as region, flock information regarding the SG9R cases are described in Table 1. Within our dataset, the number of SG cases in 2019 decreased from 37 cases and down to 10 cases in 2021. After 2021, the number of SG cases increased marginally, up to 18 cases in 2023, before decreasing to 3 cases in 2025. In contrast, the number of SG9R cases increased modestly, starting from 2019 with 1 case, and up to 9 cases annually in 2025, with SG9R cases eventually exceeding the number of SG cases submitted within the same year. Because these cases were derived from passive diagnostic submissions to a single diagnostic laboratory, the annual case numbers should be interpreted as trends within this dataset rather than as national prevalence or incidence.

**Table 1:** Summary of SG suspected field cases submitted to Avinext LTD for necropsy between 2019 ~ 2025.

No.	Case and strain no.	Location	Date	Species	Age/week-day	Mortality/day*	Gross lesions	Co-infection
1	ADL251522	Chungbuk	25.10.30	Layer	14w-6d	n/a	White foci on liver	EC^1^
2	ADL251321	Gyeonggi	25.09.22	Layer	59w-4d	n/a	Hepatomegaly, Petechiae	n/a
3	ADL251179	Gyeonggi	25.09.02	Layer	57w	n/a	Hepatomegaly, Petechiae	n/a
4	ADL251218	Gyeonggi	25.09.05	Layer	14w-d1	n/a	Hepatomegaly, Splenomegaly, Petechiae on liver	n/a
5	ADL251033	Gyeongbuk	25.08.05	Layer	45w	~30	Hepatomegaly, Splenomegaly, Petechiae	n/a
6	ADL250944	Gyeonggi	25.07.15	Layer	29w-5d	n/a	Hepatomegaly, Splenomegaly, Petechiae	EC
7	ADL250866	Chungnam	25.06.30	Layer	5w1-4d	300	Hepatomegaly, Splenomegaly, Petechiae	n/a
8	ADL250459	Chungbuk	25.04.10	Layer	11w-3d	19	Hepatomegaly, Splenomegaly, Petechiae	n/a
9	ADL250411	Chungnam	25.04.02	Layer	61w-4d	50+	Hepatomegaly, Splenomegaly, Petechiae	EC
10	ADL241328	Gyeonggi	24.10.10	Layer	13w-2d	30	White foci on liver, plug in larynx	FP^2^
11	ADL241327	Gyeonggi	24.10.10	Layer	13w-2d	30	White foci on liver, plug in larynx	FP
12	ADL240965	Chungnam	24.07.15	Layer	24w-4d	20	Hepatomegaly, Splenomegaly, Petechiae	n/a
13	ADL240955	Chungbuk	24.07.10	layer	15w-5d	n/a	Non-specific	n/a
14	ADL240655	Chungnam	24.05.17	Layer	7w	n/a	Non-specific	n/a
15	ADL240554	Chungbuk	24.05.02	Layer	46w	90	Hepatomegaly, Splenomegaly, Petechiae	n/a
16	ADL240511	Chungbuk	24.04.27	Layer	46w	n/a	Non-Specific	n/a
17	ADL231569	Chungnam	23.10.30	Breeder	23w	n/a	Hepatomegaly, White foci on liver	MD^3^, RE^4^
18	ADL231370	Gyeongnam	23.09.25	Layer	36w-2d	300	Non-specific	n/a
19	ADL231331	Chungnam	23.09.19	Layer	85w	10	Hepatomegaly, Splenomegaly, Petechiae	n/a
20	ADL231330	Chungnam	23.09.19	Layer	26w-1d	15	Hepatomegaly, Splenomegaly, Petechiae	n/a
21	ADL231047	Gyeonggi	23.07.25	Layer	15w-4d	60	Hepatomegaly, Splenomegaly, Petechiae	SG
22	ADL201773	Chungnam	20.11.04	Layer	59w-2d	n/a	Decrease in egg production (74%)	n/a
23	ADL201793	Chungbuk	20.11. 09	Layer	10w-6d	n/a	n/a	AI^5^, EC, cocci^6^, FP
24	ADL190145	Chungnam	19.01.08	Layer	7w	60	n/a	n/a

*Mortality data is based on information supplied by the farms. ^1^*E coli*, ^2^Fowl pox, ^3^Marek’s Disease, ^4^Recticuloendotheliosis, ^5^Avian Influenza, ^6^Coccidiosis.

### 3.2 SG9R-like isolates harbored SG-associated marker profiles in *aceE* and *rfaJ* and displayed smooth/O:9-positive phenotypes

Out of 24 SG9R suspected strains obtained from necropsy cases, 17 strains were shown to harbor SNPs at the targeted *rfaJ and aceE* consistent with SG-associated markers changes (Table 2). Specifically, 15 SG9R-like strains contained the AAC (Asn)- > ATC(Ile) substitution within the *aceE* gene. Nine SG9R-like strains contained the TAA (Stop)- > TCA(Serine) substitution in the *rfaJ* instead of the usual TAA (stop) mutation, corresponding to the restoration of the SG-associated marker at the position. One SG9R-like strain like (ADL231047) was shown to have the entire TCA/TAA site deleted completely in the *rfaJ* gene. Eight SG9R-like strains were shown to have both of their *aceE* and *rfaJ* mutations consistent with the SG associated profiles. In addition, four isolates, ADL240554, ADL240511, ADL231370, and ADL201793, contained the SG-associated *aceE* marker while the *rfaJ* TAA(Stop) site was changed to codons other than TCA(Ser), specifically TAC(Tyr), TAC(Tyr), GAA(Glu), and CTA(Leu), respectively. Two strains (ADL250866, ADL250411), which contained SG-associated makers in both the *aceE* and *rfaJ* genes were shown to have been originated from Farm B. Similarly, two strains (ADL240511, ADL240554) containing the same TAC(Tyr) substitution in the *rfaJ* site were also isolated from the same site (Farm C). Acriflavine tests revealed strains with SG-associated markers within the *aceE* and *rfaJ* gene displayed smooth strain characteristics of SG and were positive in O:9 antigen tests. All SG9R-like strains were shown to have the nine proline repeat domain, matching the SG9R associated profile of those identical to commercial SG9R vaccine strains. SG reference strains were shown to retain the virulence-associated markers in both *aceE* and *rfaJ,* along with proline repeat profiles (13 ~ 19), and were confirmed as smooth type(S) and positive for O:9antigen tests.

**Table 2:** SNP profiles and phenotypic characteristics of SG9R-confirmed isolates and SG9R vaccine strains.

No	Strain	Year isolation	Strain type^1^	Phenotype^2^ (rough vs. smooth)	O:9 Antigen testing	aceE nt 7–9^3^	rfaJ nt 1874–76^3^	spvB	rfaJ nt 17–18^3^
						AAC(Asn)^4^ - > ATC(Ile)	TAA(Stop)^4^ - > TCA(Ser)	Proline^5^ repeats	ATA(Ile)^4^ > ATG(Start)
1	9R vaccine #1	n/a	**SG9R**	**R**	**−**	A	A	9	ATA
2	9R vaccine #2	n/a	**SG9R**	**R**	**−**	A	A	9	ATA
3	9R vaccine #3	n/a	**SG9R**	**R**	**−**	A	A	9	ATA
4	9R vaccine #4	n/a	**SG9R**	**R**	**−**	A	A	9	ATA
5	ADL251522	25.10.30	**SG9R**	**S**	**+**	A	C	9	ATA
6	ADL251321^**A**^	25.09.22	**SG9R**	**S**	**+**	T	A	9	AT**G**
7	ADL251218	25.09.05	**SG9R**	**S**	**+**	T	C	9	ATA
8	ADL251179^**A**^	25.09.02	**SG9R**	**S**	**+**	T	A	9	AT**G**
9	ADL251033	25.08.05	**SG9R**	**S**	**+**	T	C	9	ATA
10	ADL250944^**A**^	25.07.15	**SG9R**	**S**	**+**	T	A	9	AT**G**
11	ADL250866^**B**^	25.06.30	**SG9R**	**S**	**+**	T	C	9	ATA
12	ADL250459	25.04.10	**SG9R**	**S**	**+**	T	C	9	ATA
13	ADL250411^**B**^	25.04.02	**SG9R**	**S**	**+**	T	C	9	ATA
14	ADL241328	24.10.10	**SG9R**	**R**	**−**	A	A	9	ATA
15	ADL241327	24.10.10	**SG9R**	**R**	**−**	A	A	9	ATA
16	ADL240965	24.07.15	**SG9R**	**S**	**+**	T	C	9	ATA
17	ADL240955	24.07.10	**SG9R**	**R**	**−**	A	A	9	ATA
18	ADL240655	24.05.17	**SG9R**	**R**	**−**	A	A	9	ATA
19	ADL240554^**C**^	24.05.02	**SG9R**	**S**	**+**	T	TAC^6^	9	ATA
20	ADL240511^**C**^	24.04.27	**SG9R**	**S**	**+**	T	TAC^6^	9	ATA
21	ADL231569	23.10.30	**SG9R**	**R**	**−**	A	A	9	ATA
22	ADL231370	23.09.25	**SG9R**	**S**	**+**	T	GAA^6^	9	ATA
23	ADL231331	23.09.19	**SG9R**	**S**	**+**	T	C	9	ATA
24	ADL231330	23.09.19	**SG9R**	**S**	**+**	T	C	9	ATA
25	ADL231047	23.07.25	**SG9R**	**S**	**+**	T	Deletion	9	ATA
26	ADL201793	20.11. 09	**SG9R**	**S**	**+**	A	CTA^6^	9	ATA
27	ADL201773	20.11.04	SG9R	**R**	**−**	A	A	9	ATA
28	ADL190145	19.01.08	**SG9R**	**R**	**−**	A	A	9	ATA
29	ADL250480	2025	***SG***	**S**	+	T	C	13	ATA
30	ADL250098	2025	***SG***	**S**	+	T	C	19	ATA
31	ADL250045	2025	***SG***	**S**	+	T	C	13	ATA

^1^Confirmed by Muliplex PCR. ^2^Confirmed by Acriflavine assays. ^3^Locations within the gene. ^4^SNP variation within their respective genes. Nucleotides marked in red and blue indicate SNPs native to SG and SG9R, respectively. Each cell represents the single nucleotide within the site and depicted with their respective colors. ^5^Represents the number of proline repeats in the spvB gene. ^6^Changes in site other than TAA- > TCA. ^7^SG strains listed for reference. A-Isolated from Farm A, B-Isolated from Farm B, C-Isolated from Farm C. *-Commercial SG9R vaccines currently being used in South Korea. Bold characters indicates type of strain and phenotype of the isolates.

### 3.3 A unique mutation was revealed near the attenuation associated marker within the *rfaJ* gene

**Figure 2:**
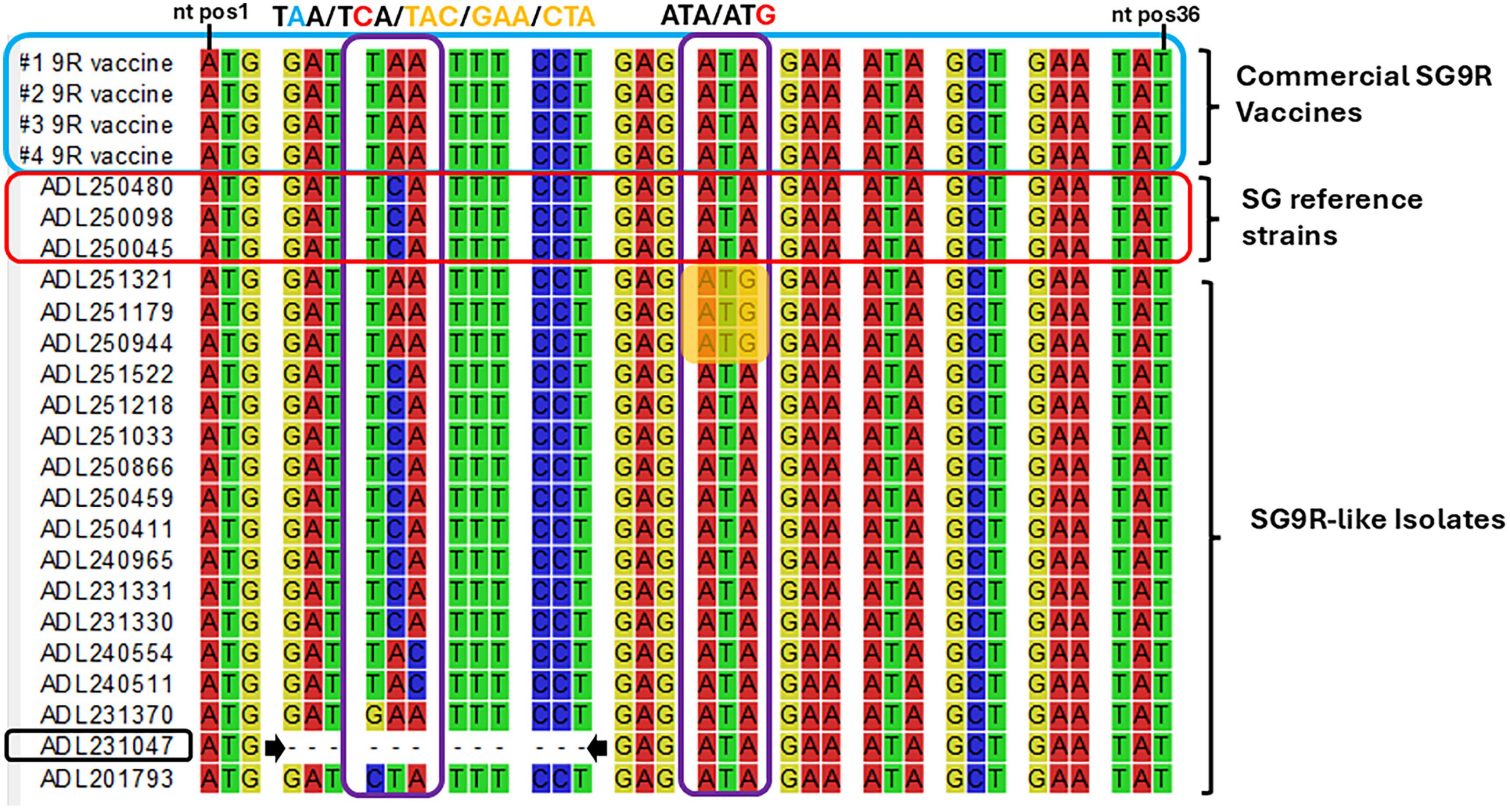
Sequence alignment of SG-associated markers and SNPs in the *rfaJ* gene of SG9R-like strains. Alignment of partial sequences of the *rfaJ* gene containing the attenuating TAA(Stop)- > TCA(Ser), GAA(Glu), TAC(Tyr), CTA(Leu) marker and the ATA(Ile)- > ATG(Start) from SG9R strains are shown. ADL250944, ADL251179, ADL251321 are SG9R-like strains from the same farm containing the ATG mutation (shaded in yellow) to allow for downstream translation of the *rfaJ* gene. In addition, the deletion of the SG-associated marker region of ADL231047 is marked in black arrows.

ATA (Ile)- > ATG(Start) mutations were identified in the *rfaJ* gene, within close proximity near the attenuation associated TAA(Stop) codon in three SG9R strains ADL250944, ADL251179 and ADL251321 (Figure 2). All strains were isolated from the same site (Farm A) but at different timepoints spanning weeks.

## 4 Discussion

Since the first documented outbreak of FT in South Korea, which occurred in 1992 (12), SG has been a long-term problem, inflicting significant economic damage to the Korean poultry industry. Prevalence of FT decreased after introduction of the SG9R vaccine in 2002 (13) but nevertheless FT is still common in the field. Despite various efforts to develop alternative SG vaccines, only vaccines based on the attenuated SG9R strain are currently licensed for commercial use in South Korea. It is well established that the *rfaJ* genes encode and are involved in the biosynthesis of the core of the LPS. Therefore, targeted analysis of *aceE* and *rfaJ* can provide useful information regarding SG-associated and SG9R-associated marker profiles, although such analysis alone cannot determine the complete genomic origin or pathogenic potential of field isolates (6, 14). Particularly, the ATC(Ser)- > TAA(Stop) substitution in the *rfaJ* gene and ATC(Ser)- > AAC(Asn) substitution in the *aceE* (4) is known as a key marker in distinguishing SG and SG9R strains. Interestingly, the SG9R-like strains which harbored at least a single SNP, consistent with SG-associated markers in the rfaJ gene, all exhibited the phenotypic characteristics of smooth type SG. These findings suggest that some SG9R-like field isolates circulating in Korea may possess marker combinations that differ from typical commercial SG9R vaccine strains and resemble SG-associated molecular and phenotypic profiles. The unique ATA(Ile)- > ATG(Start) substitution in *rfaJ* may have played a compensatory role in the disrupted *rfaJ,* by re-initiating transcription but its functional significance yet remains to be explored.

While there have been several reports regarding the reversion of SG9R strains in Europe, South America and the Africa (6–8), there has been little evidence that regular use of SG9R vaccines may lead to reversion in Korea. In our dataset, while overall number of FT cases decreased, the actual number of field cases related to the SG9R strains gradually increased, eventually exceeding the number of SG cases in 2025. Thus, the observed pattern indicates an increase in SG9R-confirmed cases within the submitted diagnostic cases analyzed in this study-however, given the nature the dataset generated in this study was based on diagnostics cases from passive surveillance, one should be cautious when intepretating the trend shown in the study.

The clinical and pathological findings warrant further investigation and attention. The isolates were obtained from actual field submissions in which chickens showed clinical signs, mortality, and gross lesions compatible with FT, including hepatomegaly, splenomegaly, hepatic petechiae, and white foci on the liver, which is common in SG. While co-infections, as shown in our dataset, may have contributed to the severity of lesions, the recovery of SG9R-like isolates with SG-associated marker profiles, smooth phenotype, and O:9 antigen positivity from clinically compatible FT cases suggests that these isolates may have field relevance. However, further investigations, such as infection studies are required to fully establish pathogenicity of these SG9R-like isolates.

What may be the driving mechanisms behind the emergence of these SG9R-like isolates? One can speculate that the long-term use of SG9R vaccines in the country, spanning nearly up to 25 years, may have played a major role. There are studies demonstrating long term use of live vaccines can be a potential factor to the genetic stability of vaccine strains in field conditions (15–17). Additionally, vaccinating flocks during onset of FT is often considered as an last-resort option in other countries to ensure non-infected birds receive protection (18). While no exact data exists on how common this type of practice is implemented in Korea, these measures may have increased opportunities for the SG9R strains and SG strains to come into contact.

Several limitations of the study exist. The isolates from the study were obtained from passive diagnostic submissions. Thus, the dataset should not be interpreted as representing the national epidemiological situation. In addition, instead of whole genome sequencing (WGS), Sanger sequencing was conducted on selected marker regions. While targeting the SG-associated markers may provide useful preliminary insight on these unique strains, it does not provide ample resolution to study phylogenetic relationships among SG, SG9R vaccine strains, and SG9R-like field isolates. Nevertheless, this study provides baseline evidence that SG9R-like isolates with SG-associated marker profiles are present among recent Korean diagnostic submissions.

To best to our knowledge, this is the first report outlining the existence of SG9R-like strains harboring SG-associated markers in South Korea. Further studies, such as whole genome sequencing (WGS), robust surveillance programs should be actively implemented to address the concerns regarding the SG landscape in the Korean poultry industry.

## Data Availability

The data presented in the study are deposited in the GenBank repository, accession numbers PZ260576~PZ260659.
